# Controlling Off-Odors in Plant Proteins Using Sequential Fermentation

**DOI:** 10.3390/foods15010039

**Published:** 2025-12-23

**Authors:** Manpreet Kaur, Charlotte Gray, Sheryl Barringer

**Affiliations:** Department of Food Science and Technology, The Ohio State University, Columbus, OH 43210, USA; kaur.333@osu.edu (M.K.); gray.1575@buckeyemail.osu.edu (C.G.)

**Keywords:** plant proteins, off-odor reduction, sequential fermentation, LAB, volatiles, SIFT-MS, deodorization, alternative dairy, clean label

## Abstract

Off-odors produced by volatile compounds remain a major barrier to consumer acceptance of plant-based proteins. This study presents a novel two-stage fermentation strategy to effectively reduce undesirable volatiles in eight plant proteins. A sequential fermentation process was developed using *Lactobacillus plantarum* in Stage 1 and a traditional yogurt culture, *Streptococcus thermophilus*, *Lactobacillus delbrueckii* subsp. *Bulgaricus* and *Lactobacillus acidophilus*, in Stage 2. This method was applied to solutions of 9% soy, pea, chickpea, mung bean, faba bean, rice, barley-rice, and hemp proteins. Volatile profiles were analyzed via Selected Ion Flow Tube Mass Spectrometry (SIFT-MS) and sensory evaluation before and after fermentation. The two-stage fermentation resulted in significant deodorization, with 95–99% reduction in key odorants such as hexanal, 2-pentylfuran, methoxypyrazines, and sulfur compounds across all proteins. The sequential approach significantly outperformed a one-stage fermentation. Allulose enhanced *L. plantarum* activity while strawberry preserves supported traditional yogurt culture performance. Non-fermentable additives such as pectin, xanthan gum, and oil had minimal effects on volatiles. The proposed fermentation method offers an effective, scalable, and clean-label solution for mitigating off-odors in plant-based proteins. By leveraging microbial metabolism and formulation synergies, this strategy provides a foundation for developing more palatable plant-based dairy alternatives.

## 1. Introduction

The global demand for sustainable, health-conscious, and allergen-friendly foods has accelerated the development of plant-based dairy alternatives, with yogurt alternatives made from plant proteins becoming increasingly popular [1]. These products offer numerous advantages, including lower environmental impact, compatibility with vegan and lactose-intolerant diets, and high nutritional value from legumes, seeds, and grains such as soy, pea, chickpea, mung bean, fava bean, hemp, rice, and barley [2]. Despite their promise, the commercial success of plant-based yogurt alternatives remains hindered by persistent issues with off-odors that negatively affect consumer acceptance [3].

These off-odors, often described as beany, grassy, earthy, sulfurous, or cereal-like, originate from a diverse array of volatile compounds that are either inherent to plant materials during cultivation or formed during processing, extraction, or storage and are influenced by both enzymatic and non-enzymatic pathways [3]. Among the volatile compounds responsible are aldehydes (e.g., hexanal, pentanal, nonanal), alcohols (e.g., 1-hexanol, 1-octen-3-ol), ketones (e.g., 2-heptanone), furans (e.g., 2-pentylfuran), pyrazines, and sulfur-containing volatiles (e.g., dimethyl disulfide, dimethyl trisulfide, hydrogen sulfide) [4].

Each plant protein contributes its own specific profile of off-odors. Soy and pea proteins are commonly associated with aldehydes such as hexanal, heptanal, nonanal, and pentanal, primarily derived from oxidative degradation of unsaturated fatty acids via the lipoxygenase pathway [4]. These aldehydes impart strong green, grassy, and fatty odors [4]. Chickpea, mung bean, and fava bean proteins also contain these aldehydes, but additionally produce methoxypyrazines such as 2-isobutyl-3-methoxypyrazine and 2-isopropyl-3-methoxypyrazine, which contribute earthy, pea-like, and pungent off-odors [4,5]. Hemp protein, due to its high lipid and sulfur amino acid content, often releases 1-octen-3-ol, 1-octen-3-one, octanal, decanal, and sulfurous compounds such as dimethyl trisulfide and hydrogen sulfide, resulting in musty, mushroom-like, or burnt rubber odors [4,5]. Rice proteins, while generally more neutral, still contribute off-notes from 1-hexanol, which can smell starchy, sweet-green, or cereal-like [5,6]. In addition to aldehydes and alcohols, other problematic volatiles include nitrogen and sulfur compounds, which are typically formed through amino acid degradation [4,5].

Extensive efforts have been made to mitigate these off-odors, ranging from breeding low-lipoxygenase varieties to thermal and chemical treatments [4]. However, these approaches often fall short. Thermal processing can denature proteins and alter texture, while chemical extraction may strip nutritional components or violate clean label expectations [4]. Most critically, these methods often target only a subset of volatiles or fail to eliminate bound compounds that release unpleasant aromas during storage or rehydration [4]. Fermentation has emerged as a natural and multifunctional strategy to reduce off-odor volatiles while enhancing sensory properties and probiotic value [4,7,8]. Lactic acid bacteria (LAB) are known for metabolizing undesirable aldehydes, sulfur volatiles, and short-chain amines into less odorous compounds or masking them with the production of pleasant acidic or fruity notes [8]. However, previous studies have typically relied on single-stage fermentation, with different lactic acid bacteria [8,9,10,11]. While effective in some cases, this approach may lack the enzymatic diversity needed to neutralize a broad spectrum of off-odor-producing volatile compounds, as each bacterial strain produces a distinct set of enzymes that target specific volatile compounds.

The potential of a sequential fermentation strategy, employing different microbial cultures at different stages to broaden deodorization capacity, remains underexplored. To address this gap, the present study investigates a two-stage microbial fermentation approach designed to reduce off-odor volatiles across a range of plant protein bases. In the first stage, *L. plantarum* is employed for its capacity to degrade aldehydes, amines, and sulfur-containing compounds. In the second stage, a traditional yogurt starter culture comprising *Streptococcus thermophilus*, *Lactobacillus delbrueckii* subsp. *bulgaricus*, and *Lactobacillus acidophilus* are added to further modulate aroma and develop desirable yogurt-like sensory characteristics. This combination leverages the complementary metabolic activities of both microbial groups by the robust deodorizing potential of *L. plantarum*, followed by the flavor-enhancing and odor-reducing functions of the standard yogurt cultures. We hypothesize that this sequential fermentation will be more effective at reducing a broad spectrum of off-odor volatile compounds in plant proteins. Sequential fermentation will work better than fermentation with both cultures co-inoculated in a single-step fermentation. This improved efficacy is expected due to the stepwise deodorization of off-odor-producing volatile compounds.

To evaluate the effectiveness of this method, key volatile compounds representing alcohols, aldehydes, ketones, sulfur volatiles, amines, pyrazines, and furans were quantified in yogurt alternatives produced from soy, pea, fava bean, chickpea, mung bean, hemp, rice, and barley-rice protein bases through sequential fermentation. The findings aim to demonstrate that sequential fermentation can serve as an effective strategy for deodorizing plant protein off-odors.

## 2. Materials and Methods

### 2.1. Preparation and Hydration of Plant Protein Solution

Plant proteins including pea (ProdEIM PEA 7028, Kerry Group, Sevilla, Spain) and rice (ProdEIM RICE 5020, Kerry Group, Sevilla, Spain), soy (ADM, Decatur, IL, USA), barley–rice (EverPro™, EverGrain Ingredients, St. Louis, MO, USA), mung bean (MB80C, Yantai T.Full Biotech Co., Ltd., Yantai, China), chickpea (CK80B, Yantai T.Full Biotech Co., Ltd., Yantai, China) and fava bean (FB90B, Yantai T.Full Biotech Co., Ltd., Yantai, China), and hemp protein (PurHP 75, Applied Food Sciences, Kerrville, TX, USA) were used in this study. To prepare the base mixture, plant protein powder was mixed with stabilizers (xanthan gum (Xan-80, AEP Colloids, Hadley, NY, USA) and/or low-methoxy pectin (GENU^®^ pectin type LM-101 AS, CPKelco, Lille Skensved, Denmark)) and allulose (It’s Just!™, Farmhouse Creative Foods, Fremont, CA, USA and Simple Truth™, The Kroger Co., Cincinnati, OH, USA) to ensure uniform distribution of all dry ingredients. This dry blend was gradually added to distilled water preheated to 55–60 °C, followed by high-shear blending using a high-speed blender (Ninja^®^ Professional 1100 W, SharkNinja Operating LLC, Needham, MA, USA) for 1 min to promote rapid dispersion and prevent clumping. During this blending step, 3% (*w*/*v*) extra virgin olive oil (Kroger™, The Kroger Co., Cincinnati, OH, USA) was also incorporated. The resulting mixture, containing 9% (*w*/*v*) plant protein and 10% (*w*/*v*) allulose, was then transferred to a beaker and maintained at 60 °C for 60 min on a magnetic hot plate stirrer (Guardian™ 5000, OHAUS Corporation, Parsippany, NJ, USA) set to 1100 rpm. Stabilizer concentrations were optimized by protein type, as shown in Table 1:

**Table 1:** Stabilizer composition by protein source.

Proteins	Xanthan Gum % (*w*/*v*)	LM Pectin % (*w*/*v*)	Calcium Chloride% (*w*/*v*)
Soy, Pea, Fava Bean, Chickpea	0.15	-	-
Hemp, Rice protein, Barley rice, Mung bean	0.30	0.67	0.04

### 2.2. Pasteurization of the Plant Protein Solution

Following the 60-min hydration step, the protein solution was subjected to pasteurization to eliminate microbial contaminants and deactivate native enzymes that could interfere with fermentation. The solution was gradually heated to 72 °C over 20 min using the heating function of a magnetic hot plate stirrer (Guardian™ 5000, OHAUS Corporation, Parsippany, NJ, USA), while maintaining continuous stirring at 1100 rpm to ensure uniform temperature distribution and prevent localized overheating. Once the solution reached 72 °C, it was held at this temperature for 15 s to complete pasteurization. Immediately following pasteurization, the mixture was cooled to 50 °C, and for formulations containing low-methoxy pectin, 0.01% (*w*/*v*) calcium chloride (CaCl_2_·2H_2_O; ACS Certified, Fisher Scientific, Fair Lawn, NJ, USA; Cat. No. C79-500) was added to initiate controlled gelation. Stirring was maintained during this step to ensure uniform calcium distribution and pectin activation without clumping.

#### 2.2.1. Fermentation Stage 1—With Lactobacillus Plantarum

Following pasteurization, the mixture was cooled to 37 °C using a cold-water bath to initiate the first fermentation stage. Freeze-dried *Lactobacillus plantarum* (strain PRBT-022; Creative Enzymes, Shirley, NY, USA) was rehydrated in sterile lukewarm water and allowed to stand for 10 min with gentle stirring. A final concentration of 0.5% (*w*/*v*) was achieved upon addition to the protein solution.

#### 2.2.2. Fermentation Setup and Incubation Conditions

The prepared plant protein solution was transferred into a sterile glass beaker for the first-stage fermentation. To minimize heat loss during incubation, the outer surface of the beaker was wrapped with aluminum foil layered with absorbent tissue paper, functioning as thermal insulation. The beaker was sealed with a layer of parafilm, and a sterile needle was inserted through the film and left in place to allow the controlled release of volatile compounds generated during fermentation. A thermometer probe was also inserted through the film to enable continuous monitoring and control of the internal temperature, which was maintained at 37 ± 1 °C. A PTFE-coated oval magnetic stir bar was used to gently agitate the viscous mixture at 500 rpm. This level of mixing ensured uniform acidification and homogeneity without introducing excess aeration, thus providing optimal conditions for the growth and metabolic activity of *Lactobacillus plantarum*. The mixture was incubated at 37 °C for 12 h under continuous stirring to ensure uniform exposure of the microbial cells to the substrate and promote effective deodorization.

### 2.3. Blending with Strawberry Preserve

Upon completion of the stage 1 fermentation step with *Lactobacillus plantarum*, 10% (*w*/*v*) strawberry preserve (Kroger™, The Kroger Co., Cincinnati, OH, USA) was added to approximately 20% of the stage 1 fermented mixture. This portion was blended in four cycles of 30 s blending followed by 30 s resting to ensure thorough dispersion of the preserve while minimizing heat generation. The blending was intentionally limited to a small portion of the mixture to reduce the risk of temperature-induced damage to the bacterial culture. Even if localized heating occurred during blending, only the blended 20% would be affected, while the remaining 80% of the stage 1 fermented mixture would retain a viable population of *L. plantarum*.

#### 2.3.1. Fermentation Stage 2—With Yogurt Cultures—*Streptococcus thermophilus*, *Lactobacillus delbrueckii* subsp. *bulgaricus*, and *Lactobacillus acidophilus*

Following recombination of the blended and unblended fractions, the second fermentation stage was initiated by adding a rehydrated commercial freeze-dried yogurt starter culture (Yogourmet^®^, Product of France; imported by C.A.P.Y.B.A.R.A Distributors Inc., Calgary, AB, Canada) containing *Streptococcus thermophilus*, *Lactobacillus delbrueckii* subsp. *bulgaricus*, and *Lactobacillus acidophilus*. The freeze-dried culture was rehydrated in sterile lukewarm distilled water and rested for 10 min with gentle agitation before being added at a final concentration of 0.83% (*w*/*v*).

#### 2.3.2. Incubation of Second Fermentation

The pre-fermented protein-strawberry preserve mixture was transferred into the container and sealed with its original tight-fitting lid, which helped maintain a controlled environment during incubation. The samples were incubated under static conditions at 37 ± 1 °C for 8 h (Soy, Pea, Fava Bean, Chickpea proteins) or 12 h (Hemp, Rice protein, Barley rice, Mung bean proteins) using a temperature-controlled yogurt maker (ULTIMATE™, Wilton, CT, USA), to support optimal texture development and controlled flavor formation.

### 2.4. Post-Fermentation Cooling

Following fermentation, the samples were first cooled at room temperature (approximately 25 °C) for 15 min to gradually transition from incubation temperature. This was followed by freezing at −18 °C for 30 min to halt microbial activity. The samples were then transferred to refrigeration at 4 °C and held for ~12 h to allow complete stabilization of the gel matrix.

### 2.5. Effect of the Ingredients on the Deodorization of Off-Odor Producing Volatiles in Pea Protein

To evaluate the contribution of each ingredient used in the formulation of the protein solution on deodorization, volatile concentrations were measured in the base protein solution and compared across treatments containing each ingredient individually. The ingredients included *L. plantarum* with allulose, *L. plantarum* with strawberry preserve, traditional yogurt cultures with allulose, traditional yogurt cultures with strawberry preserve, oil, xanthan gum, and pectin. Each treatment was prepared using the same sample quantities as those used in the sequential fermentation formulation and processed under similar conditions to ensure comparability.

### 2.6. Effect of Co-Fermentation Versus Sequential Fermentation on the Deodorization of Off-Odor Producing Volatiles in Different Plant Proteins

All steps for sample preparation were the same as described previously for the protein solution, including hydration, heating, and ingredient incorporation. Pea protein was used as the representative protein for this comparison. The only variation between treatments was the fermentation approach. For the sequential fermentation, the sample was first fermented with *L. plantarum* for 12 h, followed by the addition of strawberry preserve and inoculation with traditional yogurt cultures (*Streptococcus thermophilus* and *Lactobacillus delbrueckii* subsp. *bulgaricus*) for 8 h under similar conditions as explained in Section 2.1. For the co-fermentation, all ingredients were added to the pea protein solution simultaneously, followed by inoculation with *L. plantarum* and the traditional yogurt cultures at the same time. The mixture was then fermented for 20 h under similar conditions.

### 2.7. SIFT-MS Headspace Analysis

A 100 mL sample from each protein solution or its fermented yogurt was placed in a 500 mL Pyrex bottle. The bottle was sealed using an open-top septum-lined cap. Samples were equilibrated at room temperature for 30 min to allow headspace volatiles to stabilize. Volatile compounds (Table 2) were analyzed using Selected Ion Flow Tube–Mass Spectrometry (SIFT-MS) (Voice200ultra, Syft Technologies, Christchurch, New Zealand). Analyses were conducted in Selected Ion Mode (SIM), employing precursor ions H_3_O^+^, NO^+^, and O_2_^+^. Quantification of volatile compounds was performed using known reaction rate coefficients for ion–molecule reactions. Calibration of the instrument was conducted using a certified gas standard containing benzene, ethylbenzene, toluene, and xylene isomers. The instrument’s response was validated against known concentrations to ensure accuracy prior to sample analysis. During the test, a 14-gauge passivated needle was used to pierce the septum for sampling, with the inlet temperature maintained at 175 °C. Each sample was analyzed over a 120-s run. Three replicates were analyzed per sample type. Background levels were determined using an empty Pyrex bottle as a blank.

### 2.8. Sensory Evaluation

Based on Gacula and Rutenbeck (2006) [12], 42 untrained consumer panelists were recruited for the sensory study. The panelists consisted of students at The Ohio State University. Participants were screened to ensure they did not have allergies to plant proteins and were free from known gustatory, severe vision, or olfactory deficits and had refrained from smoking for at least two hours prior to the start of the experiment.

Samples consisted of 20 g of fermented or non-fermented protein samples and were evaluated at room temperature. Samples were presented in 100 mL Pyrex bottles with a lid and wrapped in aluminum foil to prevent the color of the sample from affecting the panelist’s feedback. The sample was labeled with random three-digit blinding codes and served in a fully randomized order to each participant. The experiments were conducted with a within-subjects design wherein each panelist served as his/her own control.

The samples were non-fermented pea protein, non-fermented soy protein, fermented pea protein, and fermented soy protein. Panelists were instructed to evaluate aroma only (no tasting) and to pause briefly between samples to minimize sensory fatigue. Participants evaluated four aroma attributes. Aroma liking was assessed on a 9-point hedonic scale from 1 = Dislike extremely to 9 = Like extremely. Aroma intensity and off-odor intensity were assessed on a 7-point descriptive scale from 1 = not perceptible to 7 = extremely strong. Panelists were also asked to rank the samples according to aroma preference from 1 = most preferred to 4 = least preferred.

All participants gave written informed consent prior to participation. This study was reviewed and approved by the Ohio State University Institutional Review Board (Study Number 20251170).

### 2.9. Statistical Analysis

All statistical analyses were conducted using JMP^®^ Pro Version 16.0.0 (Statistical Discovery, Cary, NC, USA). Graphical representations were generated using MATLAB^®^ R2024b Update 5 (Version 24.2.0.2863752, MathWorks, Natick, MA, USA). A one-way ANOVA was performed for each protein to compare volatile concentrations between fermented and non-fermented samples, as well as to evaluate the effects of individual ingredients and co-fermentation versus sequential fermentation treatments. Post hoc comparisons were performed using Fisher’s Least Significant Difference (LSD) test, with statistical significance established at *p* ≤ 0.05. All analyses were based on triplicate samples (*n* = 3) for each tested factor. For sensory data, way ANOVA followed by Fisher’s Least Significant Difference (LSD) test, with statistical significance established at *p* ≤ 0.0001.

## 3. Results

### 3.1. Effect of Sequential Fermentation on the Deodorization of Off-Odor Producing Volatiles in Different Plant Proteins

**Figure 1:**
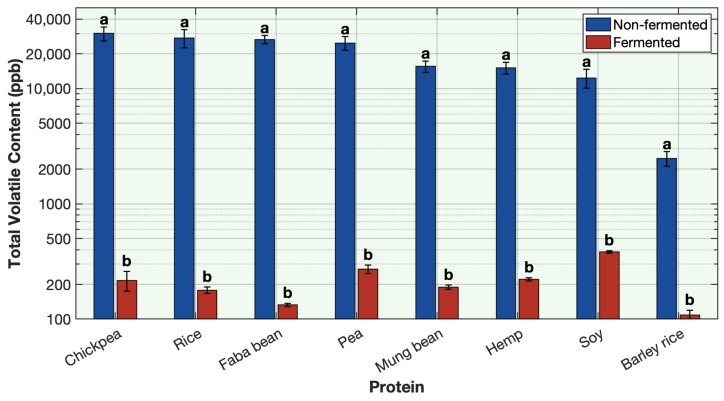
Effect of sequential fermentation on the total volatile content of different protein solutions. Volatile concentration is plotted on a base-10 logarithmic (log10) scale. Different letters indicate significant differences (*p* < 0.05) within each protein.

Volatile profiles were analyzed across eight different plant protein solutions—chickpea, rice, faba bean, pea, mung bean, hemp, soy, and barley-rice before and after sequential fermentation. A total of 31 volatiles responsible for off-odors in plant proteins were quantified in each protein and its fermented product. Across all eight proteins, sequential fermentation showed a significant reduction in the overall concentration of off-odor volatiles (Figure 1; Table A1, Table A2, Table A3, Table A4, Table A5, Table A6, Table A7 and Table A8).

Prior to fermentation, total off-odor volatile content ranged from ~26,000–30,000 ppb in pea, chickpea, rice, and faba bean proteins, ~14,000–16,000 ppb in soy, hemp, and mung bean proteins, and ~2600 ppb in barley–rice protein (Figure 1; Table A1, Table A2, Table A3, Table A4, Table A5, Table A6, Table A7 and Table A8). After fermentation, the concentration of total off-odor volatiles significantly reduced in all the proteins, to a narrow range of 185–451 ppb, representing > 98–99% overall reduction (Figure 1; Table A1, Table A2, Table A3, Table A4, Table A5, Table A6, Table A7 and Table A8). Across all the proteins, all 31 volatiles decreased significantly after fermentation (Table A1, Table A2, Table A3, Table A4, Table A5, Table A6, Table A7 and Table A8). In non-fermented proteins, aldehydes were the most abundant volatile class, followed by alcohols, with sulfur-containing compounds also present at elevated levels, while ketones, furans, and nitrogen-containing compounds were detected at comparatively lower concentrations (Table A1, Table A2, Table A3, Table A4, Table A5, Table A6, Table A7 and Table A8). All volatile classes decreased significantly, with the greatest reduction observed in aldehydes, alcohols, and sulfur-containing volatiles (Table A1, Table A2, Table A3, Table A4, Table A5, Table A6, Table A7 and Table A8). This demonstrates that the fermentation process was uniformly effective across all proteins, with differences in reduction percentages primarily reflecting initial concentration differences rather than variations in fermentation efficacy.

Pea protein was selected as a representative protein for the other proteins because it contained all major classes of off-odor volatile compounds covered in other proteins, and its deodorization pattern was consistent across all tested proteins.

**Figure 2:**
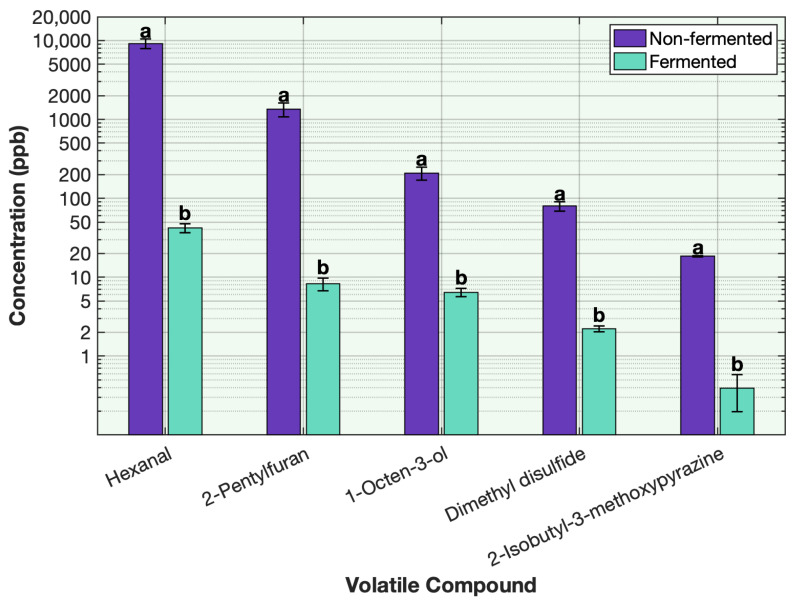
Effect of sequential fermentation on the key off-odor volatile compounds in pea protein. Volatile concentration is plotted on a base-10 logarithmic (log10) scale. Different letters indicate significant differences within each volatile (*p* < 0.05).

Among the key off-odor compounds, hexanal was the most abundant in all unfermented protein samples (Figure 2; Table A1, Table A2, Table A3, Table A4, Table A5, Table A6, Table A7 and Table A8). In pea protein, its concentration dropped dramatically from 9184 to 42 ppb following fermentation, representing > 99% reduction (Figure 2; Table A1). The final concentration falls within the reported sensory threshold range of 25–97 ppb, indicating that hexanal would be barely perceptible and that the dominant green, fatty odor note was effectively deodorized. A comparable deodorization trend was observed for 2-pentylfuran, a major beany compound originating from lipid oxidation. Its concentration declined from 1346 to 8 ppb, approaching the reported sensory threshold of 6 ppb, confirming successful suppression of its characteristic beany odor. The alcohol 1-octen-3-ol, formed by oxidation and responsible for earthy and mushroom-like notes, was also efficiently removed, decreasing from 209 to 6 ppb. As this final concentration lies within the reported sensory threshold range (0.048–10 ppb), the compound was also effectively deodorized. For nitrogenous volatiles, 2-isobutyl-3-methoxypyrazine (IBMP) decreased markedly from 18 to 0.39 ppb (>97% reduction; Figure 2; Table A1). Despite remaining above its extremely low threshold (≈0.005 ppb), this decrease represents a substantial reduction in the characteristic beany intensity. The sulfur-derived compound dimethyl disulfide (DMDS), associated with onion- and sulfur-like odors, was reduced from 79 to 2 ppb (>97% decrease), bringing its concentration well below the reported sensory threshold range of 7–12 ppb and further contributing to the overall deodorization effect.

The strong deodorization pattern seen in pea protein, characterized by a significant decrease in the concentration of hexanal, 2-pentylfuran, 1-octen-3-ol, methoxypyrazines, sulfur compounds, and other off-odors, was also evident in all of the other proteins (Table A1, Table A2, Table A3, Table A4, Table A5, Table A6, Table A7 and Table A8), confirming the broad efficacy of the fermentation process. This significant decrease in all off-odor volatile compounds can be attributed to multiple biochemical and physicochemical processes acting synergistically across the two-stage fermentation process. These mechanisms include (1) enzymatic transformation of aldehydes, ketones, sulfur, and nitrogenous volatiles by lactic acid bacteria, (2) modulation of redox potential and pH which suppresses new off odor volatile compound formation and chemical conversion of existing off odor volatile compounds, and (3) matrix-level restructuring and physical stripping that accelerate volatile release, conversion, and removal from the product.

Before fermentation begins, the plant protein powders are hydrated, homogenized, and gently heated to 60 °C to fully solubilize the proteins. These steps are essential to help release physically trapped volatiles from within the protein, making them accessible for microbial transformation during fermentation [4,5].

Once inoculated in stage one, *L. plantarum* immediately begins consuming dissolved oxygen and metabolizing available sugars, rapidly lowering the redox potential of the protein solution [13,14,15,16]. The depletion of oxygen is critical, as it stops ongoing lipid oxidation and prevents the formation of new aldehydes and ketones during fermentation [13,16]. Simultaneously, *L. plantarum* produces alcohol and aldehyde dehydrogenases that convert existing aldehydes into either less volatile alcohols or their corresponding carboxylic acids [13,14,15,16]. *L. plantarum* also produces low levels of hydrogen peroxide via NADH oxidase activity, which creates a mildly oxidative environment that converts reactive sulfur thiols into disulfides or sulfoxides with significantly higher odor thresholds [13,14,15,16]. As fermentation progresses, a moderate decrease in pH (to ~5.0) begins to alter the surface charge of the protein matrix [17], leading to the partial desorption and increased mobility of previously surface-bound volatile compounds [18]. Continuous stirring and controlled venting further facilitate the physical stripping of these released volatiles from the headspace. By the end of stage 1, aldehydes, alcohols, ketones, furans, sulfur compounds, and methoxypyrazines have already been reduced through combined enzymatic conversion, controlled oxidation, early acid-induced release, and physical removal.

In the second stage, inoculation with yogurt starter cultures (*Streptococcus thermophilus*, *Lactobacillus delbrueckii* subsp. *bulgaricus*, and *L. acidophilus*) triggers rapid lactic acid production, which reduces the pH to approximately 4.5 within 8–12 h [19,20]. This acidification is critical for multiple deodorization mechanisms. First, it suppresses further lipid oxidation by scavenging oxygen-reactive radicals such as hydroxyl radicals and hydrogen peroxide and removing secondary lipid-oxidation products, preventing propagation of oxidative damage [21]. Second, basic nitrogen-containing volatiles such as amines and methoxypyrazines undergo protonation, converting into charged, non-volatile forms with negligible vapor pressure and sensory impact [22,23]. Third, the acidic environment drives protein unfolding and gelation near the isoelectric point, leading to aggregation, and expulsion of water and any residual free volatiles from the matrix [24,25,26,27]. This unfolding process also exposes additional thiol and amino functional groups capable of covalently binding aldehydes and sulfur compounds, further reducing their volatility [24,25,26,27]. As gelation progresses, the forming three-dimensional protein network physically traps trace off-odor volatiles [28,29], preventing their release into the headspace. Together, these biochemical and structural transformations complete the deodorization initiated in Stage 1.

### 3.2. Sensory Evaluation of Sequential Fermentation on the Deodorization of Off-Odor Producing Volatiles in Different Plant Proteins

**Figure 3:**
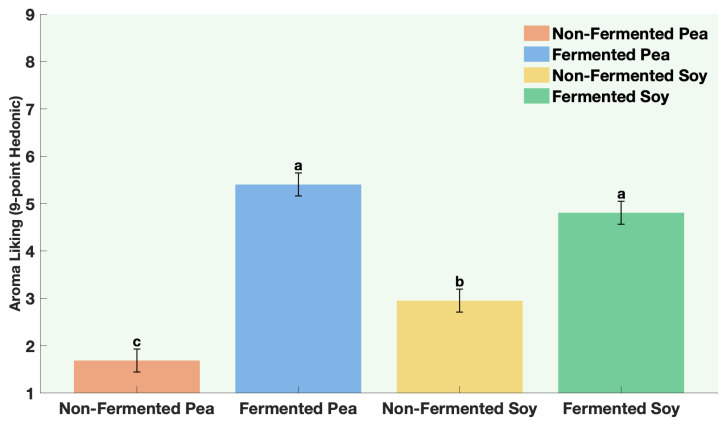
Aroma liking of non-fermented vs. sequentially-fermented protein. Different letters indicate significant differences (*p* < 0.0001).

Sensory evaluation results supported the SIFT-MS headspace analysis, confirming the positive impact of fermentation on aroma quality. Fermentation significantly improved aroma liking for both pea and soy proteins (Figure 3, Table A11). Non-fermented pea protein received the lowest liking scores, indicating strong aroma rejection by consumers (Figure 3, Table A11). Fermented pea protein showed a substantial increase in liking (Figure 3, Table A11), demonstrating effective sensory improvement through sequential fermentation. A similar trend was observed for soy, where sequential fermentation led to a significant increase in liking compared to the non-fermented soy sample (Figure 3). These findings confirm that fermentation enhanced overall sensory acceptability by improving the perceived aroma quality of the protein samples.

**Figure 4:**
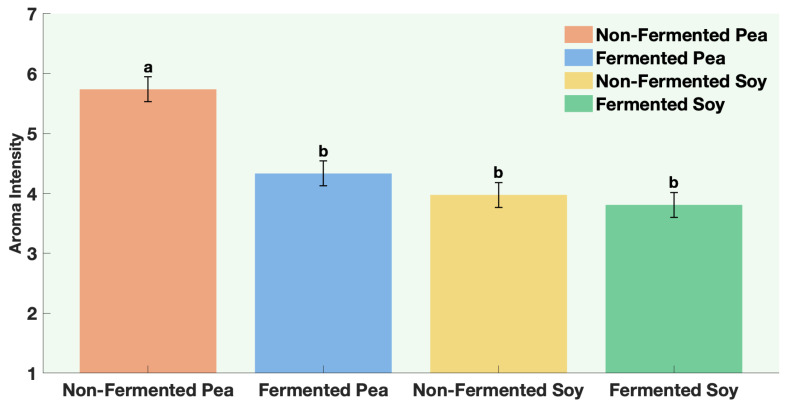
Aroma Intensity of non-fermented vs. sequentially-fermented protein. Different letters indicate significant differences (*p* < 0.0001).

**Figure 5:**
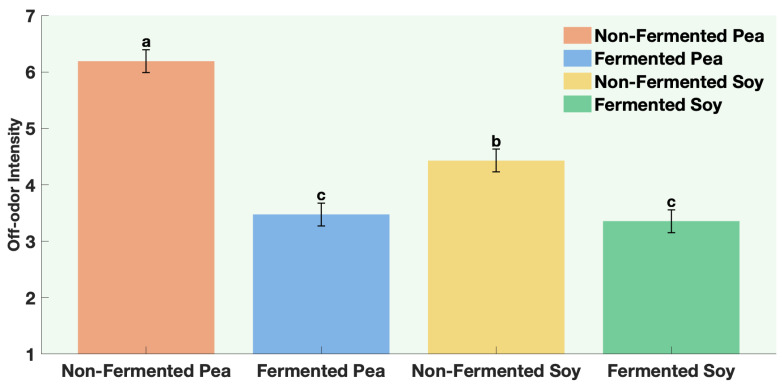
Off-Odor Intensity of non-fermented vs. sequentially-fermented protein. Different letters indicate significant differences (*p* < 0.05).

Overall aroma intensity decreased when the pea was fermented, but remained unchanged for soy (Figure 4, Table A11). However, off-odor intensity was significantly higher in the non-fermented pea and soy samples, while fermented samples showed clearly lower off-odor perception (Figure 5, Table A11). This indicates that sequential fermentation improved the overall aroma by lowering off-odors.

Samples were further evaluated using a ranking test, where panelists ranked the four samples from 1, indicating “most preferred”, to 4, indicating “least preferred”, based on aroma perception. Lower mean rank indicates higher preference. The ranking results showed that fermented samples were ranked significantly more favorably than their non-fermented counterparts (Table 3). The ranking trend closely matched both the aroma liking and off-odor intensity results, reinforcing that fermentation improved consumer preference by effectively reducing objectionable odor notes.

### 3.3. Effect of the Ingredients on the Deodorization of Off-Odor Volatile Compounds in Pea Protein

**Figure 6:**
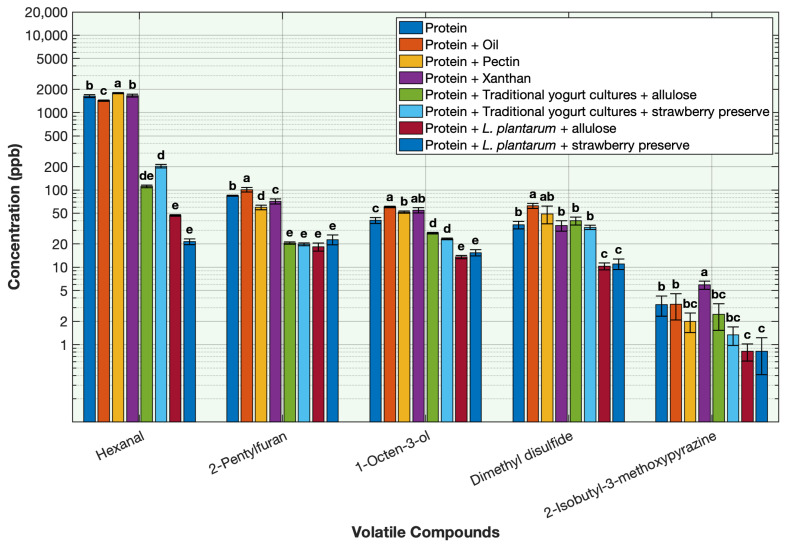
Effect of different ingredients used in the fermentation on the key off-odor volatile compounds in pea protein. Volatile concentration is plotted on a base-10 logarithmic (log10) scale. Different letters indicate significant differences within each volatile (*p* < 0.05).

To evaluate the contribution of each component on deodorization, volatile concentrations were measured in the protein solution and compared to protein samples with the addition of oil, pectin, xanthan gum, yogurt standard culture with allulose, yogurt standard culture with strawberry preserve, *Lactobacillus plantarum* with allulose, and *L. plantarum* with strawberry preserve (Figure 6, Table A9). The protein solution with lactic acid bacterial cultures produced the greatest deodorization, whereas viscosifying ingredients such as xanthan gum, pectin, and oil had a minimal effect on the volatiles.

Fermentation with *L. plantarum* significantly decreased all the off-odor volatiles, with most volatiles reduced by over 90% compared to the unfermented protein solution (Figure 6, Table A9). Concentrations of hexanal, 2-pentylfuran, 1-octen-3-ol, dimethyl disulfide, and 2-isobutyl-3-methoxypyrazine were significantly lower than in the unfermented protein solution, confirming the strong deodorizing potential of this culture. The extensive volatile reduction is consistent with *L. plantarum*’s ability to metabolize aldehydes, alcohols, and sulfur compounds, as explained in Section 3.1 [13,14,15,16,17,18].

Carbohydrate substrate also influenced the overall pattern of deodorization. *L. plantarum* paired with allulose produced a greater reduction of 1-octen-3-ol, dimethyl disulfide, and 2-pentylfuran compared to its pairing with strawberry preserve (Figure 6). This pattern suggests that allulose supports *L. plantarum*’s metabolism and redox-driven degradation of off-odor volatile compounds. In comparison, yogurt starter cultures appeared to perform better with strawberry preserve, showing lower concentrations of most volatiles relative to the allulose. This trend likely reflects faster acidification and enhanced volatilization caused by the presence of simple sugars and organic acids in the fruit preserve. Although both sugars were effective in supporting fermentation, these results indicate that metabolic compatibility between the culture and its carbon source can influence deodorization efficiency.

Fermentation with traditional yogurt cultures (*Streptococcus thermophilus* and *Lactobacillus delbrueckii* subsp. *bulgaricus*) decreased off-odor volatiles, though to a lesser extent than *L. plantarum*. The rapid acidification explained in Section 3.1 during stage 2 (final pH ~ 4.5) likely contributed to the volatile reduction [19,20,21,22,23,24,25,26,27,28,29].

The addition of oil showed minimal reduction in a few volatiles through formation of a hydrophobic microphase in oil that temporarily solubilizes nonpolar volatiles, thereby lowering their apparent headspace concentration [26,30]. The hydrocolloid stabilizers xanthan gum and pectin also had a limited impact on deodorization and showed minimal reduction in a few volatiles through entrapment of off-odor volatiles within the hydrocolloid network through hydrophobic or hydrogen bonds [31,32].

### 3.4. Effect of Co-Fermentation Versus Sequential Fermentation on the Deodorization of Off-Odor Producing Volatiles in Pea Protein

**Figure 7:**
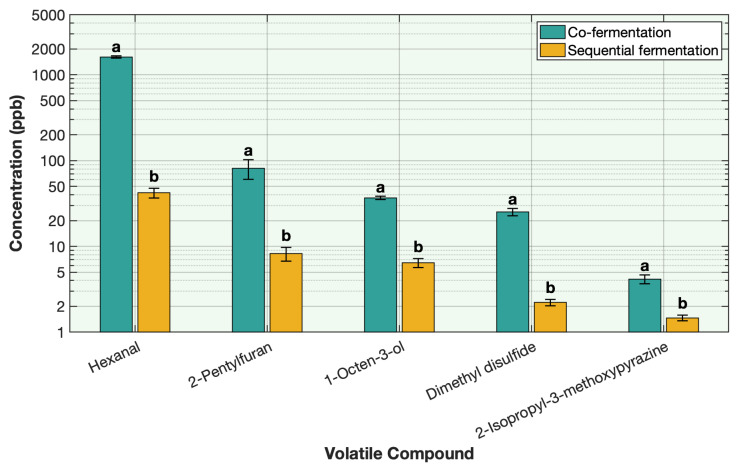
Effect of one stage vs. sequential fermentation on the key off-odor volatile compounds in pea protein. Volatile concentration is plotted on a base-10 logarithmic (log10) scale. Different letters indicate significant differences within each volatile (*p* < 0.05).

To study the comparative efficiency of sequential fermentation versus co-fermentation, the volatile profiles of 20-h co-fermentation with both *Lactobacillus plantarum* and traditional yogurt cultures and of sequential fermentation with *L. plantarum* for 12 h followed by 8 h of yogurt culture fermentations were analyzed. Although both fermentation methods employed the same microbial strains (*Lactobacillus plantarum* and traditional yogurt cultures), the order and timing of inoculation had a pronounced effect on off-odor reduction. Across all off-odor volatiles, the sequential fermentation yielded substantially lower post-fermentation concentrations of off-odor volatiles compared to the co-fermentation (Figure 7, Table A10). Concentrations of hexanal, 2-pentylfuran, 1-octen-3-ol, dimethyl disulfide, and 2-isopropyl-3-methoxypyrazine were reduced by 97–99% under sequential fermentation, compared with 68–93% under co-fermentation (Figure 7, Table A10).

The enhanced effectiveness of the sequential fermentation is attributed to the sequential operation of *Lactobacillus plantarum* and yogurt starter cultures, as explained in Section 3.1, under their respective optimal conditions. In the first 12-h stage, *Lactobacillus plantarum* metabolized off-odor volatiles via alcohol and aldehyde dehydrogenase pathways [13,14,15,16] and consumed residual oxygen to stabilize the redox environment [13,14,15,16]. This stage proceeds at a mildly acidic pH (~5.5–6.0), which coincides with the optimal activity range of *L. plantarum* and supports efficient enzymatic redox reactions [13,14,15,16]. Because *L. plantarum* produces organic acids more slowly than traditional yogurt cultures [33,34,35], the pH remains relatively stable (between 6.0 and 5.5) during this stage, allowing its dehydrogenase enzymes to remain active and effectively metabolize off-odor volatile compounds [13,14,15,16]. Mild proteolysis during this step loosens the protein matrix, enhancing the accessibility and diffusivity of entrapped volatiles [17,18]. In the subsequent 8-h stage, yogurt cultures (*Streptococcus thermophilus*, *Lactobacillus delbrueckii* subsp. *bulgaricus*, and *L. acidophilus*) rapidly ferment the available sugars, driving the pH to ~4.5 and preventing oxidation, promoting protonation and volatilization of basic and carbonyl compounds [21,22,23,24,25,26,27]. Acid gelation of the matrix further facilitates volatile release or binding into non-volatile complexes [28,29]. Simultaneously, the yogurt bacteria generate desirable volatiles that contribute pleasant dairy-like and fruity aromas and mask any residual off-notes [36,37,38].

In co-fermentation, *Lactobacillus plantarum* and traditional yogurt cultures were inoculated simultaneously. Although the starting pH (~7) was initially suitable for both cultures, the traditional yogurt cultures rapidly acidified the medium, driving the pH below 5.5 within a short time [39]. This early acidification shortened the time during which *L. plantarum* could function within its optimal range (pH 5.5–6.0) [40], thereby limiting its enzymatic redox conversion of aldehydes and sulfur volatiles. As the two microbial groups metabolized sugars concurrently, the yogurt cultures consumed most of the available carbon sources and generated lactic acid faster than *L. plantarum* could adjust metabolically [39,40]. The resulting rapid pH decline inhibited *L. plantarum* dehydrogenase activity and also led to early protein coagulation, reducing the diffusivity of volatiles [41,42,43]. This concurrent metabolism, therefore, disrupted the balanced, stagewise progression achieved in sequential fermentation, in which *L. plantarum* first metabolizes off-odor volatiles under mildly acidic conditions before traditional yogurt cultures finalize acidification and further metabolize them. As a result, co-fermentation led to less effective deodorization than sequential fermentation.

## 4. Conclusions

This study demonstrates the remarkable effectiveness of sequential fermentation as a strategy for deodorizing plant-based proteins. By employing a two-stage sequential fermentation process initially with *Lactobacillus plantarum* followed by a traditional yogurt culture, the formulation achieved a broad and substantial reduction in off-odor volatile compounds across eight different plant proteins, including soy, pea, chickpea, mung bean, faba bean, rice, barley-rice, and hemp. The SIFT-MS headspace analysis and sensory results revealed consistent and often near-complete reductions in key off-odor volatiles such as aldehydes, alcohols, methoxypyrazines, ketones, and sulfur volatiles. Among the volatile classes, aldehydes, alcohols, and sulfur compounds showed the most dramatic decreases. This outcome is attributed to the synergistic enzymatic activities of the microbial cultures, supported by matrix acidification, redox effects, protein denaturation, and strategic venting during fermentation. The use of LAB cultures with specific sugar sources, such as allulose with *L. plantarum* and strawberry preserve with yogurt cultures, further enhanced the deodorization efficiency and contributed desirable sensory qualities to the final product. The co-fermentation approach, where all ingredients and cultures were combined simultaneously, was less effective. Although some deodorization was observed, it was not as effective as sequential fermentation. The simultaneous presence of multiple microbial strains likely led to early competition for nutrients and suboptimal enzymatic activity, highlighting the importance of sequential fermentation. This work not only advances the understanding of how fermentation can mitigate the sensory challenges associated with plant protein ingredients but also offers a practical, clean-label solution for the development of flavorful plant-based dairy alternatives. By combining microbiological expertise with food chemistry, the proposed method achieves deodorization without reliance on artificial additives. The successful application across diverse protein types reinforces the potential of this approach to be generalized in future plant-based product innovations.

## 5. Patents

Provisional Patent Number 63/898,020.

## Figures and Tables

**Table 2:** Properties of off-odor volatiles quantified in selected-ion flow tube mass spectrometer (SIFT-MS) headspace analysis.

Volatile	Reagent	k (10^−9^ cm^3^/s)	Mass (*m*/*z*)	Product
(E)-2-pentenal	NO^+^	4	83	C_5_H_7_O^+^
(E,Z)-2,6-nonadienal	NO^+^	2.5	137	C_9_H_13_O^+^
1-hexanol	NO^+^	2.4	101	C_6_H_13_O^+^
1-octanol	NO^+^	2.3	129	C_8_H_17_O^+^
1-octen-3-ol	H_3_O^+^	2.5	111	C_8_H_15_^+^
1-octen-3-one	NO^+^	2.5	156	C_8_H_14_·NO^+^
1-pentanol	H_3_O^+^	2.8	71	C_5_H_11_^+^
1-penten-3-ol	H_3_O^+^	2.6	69	C_5_H_9_^+^
2,4-decadienal	NO^+^	4.2	151	C_10_H_15_O^+^
2,4-heptadienal	NO^+^	2.1	57	C_3_H_5_O^+^
2-heptanone	NO^+^	3.4	144	C_7_H_14_O·NO^+^
2-isobutyl-3-methoxypyrazine	H_3_O^+^	3.0	167	C_9_H_14_N_2_O·H^+^
	NO^+^	1.3	124	C_6_H_10_N_2_O^+^
	O_2_^+^	1.3	124	C_6_H_8_N_2_O^+^
2-isopropyl-3-methoxypyrazine	H_3_O^+^	3	153	C_8_H_12_N_2_O·H^+^
2-octenal	NO^+^	4.1	125	C_8_H_13_O^+^
2-pentylfuran	H_3_O^+^	3.0	139	C_9_H_14_O·H^+^
	NO^+^	2.0	138	C_9_H_14_O^+^
3-hexen-1-ol	H_3_O^+^	3.2	83	C_6_H_11_^+^
Butanal	NO^+^	2.3	71	C_4_H_7_O^+^
Carbon disulfide	O_2_^+^	4	76	CS_2_^+^
Decanal	NO^+^	3.3	155	C_10_H_19_O^+^
Dimethyl disulfide	NO^+^	2.4	94	(CH_3_)_2_S_2_^+^
Dimethyl trisulfide	H_3_O^+^	2.8	145	C_2_H_6_S_3_H^+^·H_2_O^+^
	NO^+^	1.9	126	C_2_H_6_S_3_^+^
	O_2_^+^	2.2	126	C_2_H_6_S_3_^+^
Dimethylamine	H_3_O^+^	2.1	46	(CH_3_)_2_NH·H^+^
Formaldehyde	H_3_O^+^	3.4	31	CH_3_O^+^
			49	H_2_CO·H^+^·H_2_O
Heptanal	NO^+^	3.3	113	C_7_H_13_O^+^
Hexanal	NO^+^	2.5	99	C_6_H_11_O^+^
Hydrogen sulfide	H_3_O^+^	1.6	35	H_3_S^+^
Methional	H_3_O^+^	3.0	105	C_4_H_8_S·H^+^
	NO^+^	2.5	104	C_4_H_8_OS^+^
	O_2_^+^	2.5	104	C_4_H_8_OS^+^
Methyl mercaptan	H_3_O^+^	1.8	49	CH_4_S·H^+^
Nonanal	NO^+^	2.7	141	C_9_H_17_O^+^
Octanal	NO^+^	3	127	C_8_H_15_O^+^
Pentanal	NO^+^	3	85	C_5_H_9_O^+^

**Table 3:** Aroma preference ranking of non-fermented vs. sequentially-fermented protein. Different letters indicate significant differences within samples (*p* < 0.0001).

Samples	Ranking	Preference
Fermented Pea	1.6 ^c^	Most
Fermented Soy	1.8 ^c^	Most
Non-Fermented Soy	2.8 ^b^	Least
Non-Fermented Pea	3.8 ^a^	Least

## Data Availability

The original contributions presented in the study are included in the article. Further inquiries can be directed to the corresponding author.

## Appendix A

**Table A1:** Deodorization of Pea protein (PP) using sequential fermentation. Different letters indicate significant differences within each volatile (*p* < 0.05).

Volatiles	PP Non-Fermented	PP Fermented
(E)-2-pentenal	71.7 ^a^	5.32 ^b^
(E,Z)-2,6-nonadienal	11.1 ^a^	0.40 ^b^
1-hexanol	78.2 ^a^	17.7 ^b^
1-octanol	15.5 ^a^	1.51 ^b^
1-octen-3-ol	209 ^a^	6.44 ^b^
1-octen-3-one	24.9 ^a^	0.85 ^b^
1-pentanol	400 ^a^	26.4 ^b^
1-penten-3-ol	1326 ^a^	25.1 ^b^
2,4-decadienal	7.71 ^a^	0.70 ^b^
2,4-heptadienal	1232 ^a^	12.1 ^b^
2-heptanone	888 ^a^	20.6 ^b^
2-isobutyl-3-methoxypyrazine	18.4 ^a^	0.39 ^b^
2-isopropyl-3-methoxypyrazine	18.3 ^a^	1.47 ^b^
2-octenal	122 ^a^	2.58 ^b^
2-pentylfuran	1346 ^a^	8.23 ^b^
3-hexen-1-ol	4419 ^a^	26.5 ^b^
butanal	977 ^a^	20.2 ^b^
carbon disulfide	2248 ^a^	15.9 ^b^
decanal	7.57 ^a^	1.14 ^b^
dimethyl disulfide	79.5 ^a^	2.22 ^b^
dimethyl trisulfide	58.7 ^a^	1.54 ^b^
dimethylamine	161 ^a^	59.9 ^b^
formaldehyde	852 ^a^	9.72 ^b^
heptanal	211 ^a^	3.85 ^b^
hexanal	9184 ^a^	42.1 ^b^
hydrogen sulfide	161 ^a^	0.97 ^b^
methional	16.6 ^a^	0.39 ^b^
methyl mercaptan	982 ^a^	4.97 ^b^
nonanal	46.6 ^a^	1.98 ^b^
octanal	113 ^a^	3.34 ^b^
pentanal	3017 ^a^	33.1 ^b^

**Table A2:** Deodorization of Rice protein (RP) using sequential fermentation. Different letters indicate significant differences within each volatile (*p* < 0.05).

Volatiles	RP Non-Fermented	RP Fermented
(E)-2-pentenal	73.0 ^a^	1.80 ^b^
(E,Z)-2,6-nonadienal	38.7 ^a^	0.71 ^b^
1-hexanol	92.8 ^a^	6.28 ^b^
1-octanol	19.8 ^a^	1.19 ^b^
1-octen-3-ol	279 ^a^	5.48 ^b^
1-octen-3-one	62.5 ^a^	1.60 ^b^
1-pentanol	639 ^a^	15.1 ^b^
1-penten-3-ol	1175 ^a^	12.2 ^b^
2,4-decadienal	4.16 ^a^	0.47 ^a^
2,4-heptadienal	997 ^a^	10.8 ^b^
2-heptanone	713 ^a^	3.36 ^b^
2-isobutyl-3-methoxypyrazine	7.74 ^a^	0.67 ^b^
2-isopropyl-3-methoxypyrazine	12.2 ^a^	0.84 ^b^
2-octenal	75.4 ^a^	0.90 ^b^
2-pentylfuran	299 ^a^	2.28 b
3-hexen-1-ol	5594 ^a^	24.5 ^b^
butanal	973 ^a^	12.2 ^b^
carbon disulfide	2231 ^a^	8.03 ^b^
decanal	9.46 ^a^	0.81 ^b^
dimethyl disulfide	273 ^a^	1.89 ^b^
dimethyl trisulfide	92.2 ^a^	2.28 ^b^
dimethylamine	163 ^a^	74.0 ^b^
formaldehyde	237 ^a^	11.6 ^b^
heptanal	252 ^a^	2.50 ^b^
hexanal	11,630 ^a^	37.4 ^b^
hydrogen sulfide	21.7 ^a^	0.69 ^b^
methional	12.8 ^a^	0.37 ^b^
methyl mercaptan	272 ^a^	9.96 ^b^
nonanal	63.5 ^a^	2.42 ^b^
octanal	199 ^a^	2.85 ^b^
pentanal	3649 ^a^	17.7 ^b^

**Table A3:** Deodorization of Soy protein (SP) using sequential fermentation. Different letters indicate significant differences within each volatile (*p* < 0.05).

Volatiles	SP Non-Fermented	SP Fermented
(E)-2-pentenal	95.5 ^a^	6.61 ^b^
(E,Z)-2,6-nonadienal	6.44 ^a^	0.48 ^b^
1-hexanol	37.8 ^a^	26.9 ^a^
1-octanol	5.73 ^a^	1.57 ^b^
1-octen-3-ol	75.2 ^a^	5.51 ^b^
1-octen-3-one	36.1 ^a^	0.99 ^b^
1-pentanol	175 ^a^	154 ^a^
1-penten-3-ol	703 ^a^	30.6 ^b^
2,4-decadienal	1.66 ^a^	0.70 ^b^
2,4-heptadienal	1668 ^a^	12.4 ^b^
2-heptanone	92.3 ^a^	3.64 ^b^
2-isobutyl-3-methoxypyrazine	1.79 ^a^	0.49 ^a^
2-isopropyl-3-methoxypyrazine	3.78 ^a^	1.49 ^b^
2-octenal	23.0 ^a^	0.88 ^b^
2-pentylfuran	83.5 ^a^	2.10 ^b^
3-hexen-1-ol	2797 ^a^	43.3 ^b^
butanal	378 ^a^	19.6 ^b^
carbon disulfide	1181 ^a^	10.0 ^b^
decanal	4.29 ^a^	0.77 ^b^
dimethyl disulfide	7.44 ^a^	1.24 ^b^
dimethyl trisulfide	18.1 ^a^	1.06 ^b^
dimethylamine	303 ^a^	149 ^b^
formaldehyde	411 ^a^	42.5 ^b^
heptanal	85.4 ^a^	2.29 ^b^
hexanal	5388 ^a^	5.15 ^b^
hydrogen sulfide	198 ^a^	1.15 ^b^
methional	7.17 ^a^	0.64 ^b^
methyl mercaptan	494 ^a^	26.7 ^b^
nonanal	42.5 ^a^	1.33 ^b^
octanal	49.8 ^a^	2.61 ^b^
pentanal	1733 ^a^	29.7 ^b^

**Table A4:** Deodorization of Hemp protein (HP) using sequential fermentation. Different letters indicate significant differences within each volatile (*p* < 0.05).

Volatiles	HP Non-Fermented	HP Fermented
(E)-2-pentenal	246 ^a^	6.07 ^b^
(E,Z)-2,6-nonadienal	8.38 ^a^	0.40 ^b^
1-hexanol	1536 ^a^	18.7 ^b^
1-octanol	17.5 ^a^	1.59 ^b^
1-octen-3-ol	33.7 ^a^	3.67 ^b^
1-octen-3-one	72.6 ^a^	1.27 ^b^
1-pentanol	885 ^a^	16.7 ^b^
1-penten-3-ol	597 ^a^	10.8 ^b^
2,4-decadienal	1.66 ^a^	1.18 ^a^
2,4-heptadienal	2001 ^a^	10.0 ^b^
2-heptanone	106 ^a^	10.6 ^b^
2-isobutyl-3-methoxypyrazine	4.08 ^a^	0.44 ^b^
2-isopropyl-3-methoxypyrazine	3.60 ^a^	0.87 ^b^
2-octenal	21.7 ^a^	0.69 ^b^
2-pentylfuran	19.0 ^a^	2.10 ^b^
3-hexen-1-ol	2067 ^a^	34.7 ^b^
butanal	358 ^a^	5.62 ^b^
carbon disulfide	1773 ^a^	6.88 ^b^
decanal	6.31 ^a^	1.65 ^b^
dimethyl disulfide	26.7 ^a^	1.89 ^b^
dimethyl trisulfide	28.5 ^a^	1.81 ^b^
dimethylamine	154 ^a^	93.7 ^b^
formaldehyde	1111 ^a^	69.5 ^b^
heptanal	49.8 ^a^	2.20 ^b^
hexanal	4276 ^a^	13.3 ^b^
hydrogen sulfide	20.2 ^a^	4.96 ^b^
methional	5.77 ^a^	0.69 ^a^
methyl mercaptan	1294 ^a^	61.0 ^b^
nonanal	18.6 ^a^	2.23 ^b^
octanal	30.9 ^a^	1.55 ^b^
pentanal	1394 ^a^	9.54 ^b^

**Table A5:** Deodorization of Chickpea protein (CP) using sequential fermentation. Different letters indicate significant differences within each volatile (*p* < 0.05).

Volatiles	CP Non-Fermented	CP Fermented
(E)-2-pentenal	55.8 ^a^	3.93 ^b^
(E,Z)-2,6-nonadienal	9.77 ^a^	0.22 ^b^
1-hexanol	94.6 ^a^	18.9 ^b^
1-octanol	10.8 ^a^	0.88 ^b^
1-octen-3-ol	128 ^a^	3.23 ^b^
1-octen-3-one	32.7 ^a^	1.01 ^b^
1-pentanol	318 ^a^	47.5 ^b^
1-penten-3-ol	863 ^a^	11.9 ^b^
2,4-decadienal	2.58 ^a^	0.42 ^b^
2,4-heptadienal	939 ^a^	11.9 ^b^
2-heptanone	373 ^a^	4.56 ^b^
2-isobutyl-3-methoxypyrazine	3.35 ^a^	0.62 ^b^
2-isopropyl-3-methoxypyrazine	5.52 ^a^	0.59 ^b^
2-octenal	86.4 ^a^	0.63 ^b^
2-pentylfuran	541 ^a^	2.40 ^b^
3-hexen-1-ol	6835 ^a^	30.4 ^b^
butanal	622 ^a^	10.1 ^b^
carbon disulfide	1661 ^a^	5.18 ^b^
decanal	4.69 ^a^	1.01 ^b^
dimethyl disulfide	47.2 ^a^	2.62 ^b^
dimethyl trisulfide	42.4 ^a^	1.00 ^b^
dimethylamine	222 ^a^	74.9 ^b^
formaldehyde	1899 ^a^	31.2 ^b^
heptanal	180 ^a^	2.36 ^b^
hexanal	13,818 ^a^	23.1 ^b^
hydrogen sulfide	199 ^a^	0.58 ^b^
methional	13.5 ^a^	0.44 ^b^
methyl mercaptan	2251 ^a^	17.1 ^b^
nonanal	58.7 ^a^	2.47 ^b^
octanal	98.5 ^a^	2.18 ^b^
pentanal	2627 ^a^	15.4 ^b^

**Table A6:** Deodorization of Faba bean protein (FBP) using sequential fermentation. Different letters indicate significant differences within each volatile (*p* < 0.05).

Volatiles	FBP Non-Fermented	FBP Fermented
(E)-2-pentenal	51.4 ^a^	3.63 ^b^
(E,Z)-2,6-nonadienal	8.08 ^a^	0.34 ^b^
1-hexanol	87.5 ^a^	16.1 ^b^
1-octanol	8.46 ^a^	0.84 ^b^
1-octen-3-ol	134 ^a^	3.31 ^b^
1-octen-3-one	41.3 ^a^	0.78 ^b^
1-pentanol	308 ^a^	32.7 ^b^
1-penten-3-ol	798 ^a^	8.69 ^b^
2,4-decadienal	2.52 ^a^	0.52 ^b^
2,4-heptadienal	1544 ^a^	16.4 ^b^
2-heptanone	393 ^a^	4.54 ^b^
2-isobutyl-3-methoxypyrazine	5.55 ^a^	0.22 ^b^
2-isopropyl-3-methoxypyrazine	5.63 ^a^	0.79 ^b^
2-octenal	71.5 ^a^	0.50 ^b^
2-pentylfuran	459 ^a^	1.52 ^b^
3-hexen-1-ol	6175 ^a^	12.7 ^b^
butanal	610 ^a^	4.57 ^b^
carbon disulfide	1887 ^a^	4.14 ^b^
decanal	4.69 ^a^	0.47 ^b^
dimethyl disulfide	33.8 ^a^	1.18 ^b^
dimethyl trisulfide	35.7 ^a^	1.25 ^b^
dimethylamine	192 ^a^	81.1 ^b^
formaldehyde	204 ^a^	17.9 ^b^
heptanal	183 ^a^	1.81 ^b^
hexanal	12,962 ^a^	3.66 ^b^
hydrogen sulfide	41.8 ^a^	0.65 ^b^
methional	10.0 ^a^	0.25 ^b^
methyl mercaptan	236 ^a^	6.10 ^b^
nonanal	39.1 ^a^	1.90 ^b^
octanal	90.0 ^a^	1.89 ^b^
pentanal	2396 ^a^	6.05 ^b^

**Table A7:** Deodorization of Mung bean protein (MBP) using sequential fermentation. Different letters indicate significant differences within each volatile (*p* < 0.05).

Volatiles	MBP Non-Fermented	MBP Fermented
(E)-2-pentenal	78.1 ^a^	4.93 ^b^
(E,Z)-2,6-nonadienal	5.41 ^a^	0.62 ^b^
1-hexanol	40.7 ^a^	12.7 ^b^
1-octanol	5.25 ^a^	1.20 ^b^
1-octen-3-ol	88.8 ^a^	5.44 ^b^
1-octen-3-one	12.4 ^a^	1.41 ^b^
1-pentanol	215 ^a^	29.8 ^b^
1-penten-3-ol	915 ^a^	30.8 ^b^
2,4-decadienal	1.32 ^a^	0.99 ^a^
2,4-heptadienal	2775 ^a^	10.2 ^b^
2-heptanone	153 ^a^	4.88 ^b^
2-isobutyl-3-methoxypyrazine	5.56 ^a^	0.18 ^b^
2-isopropyl-3-methoxypyrazine	2.90 ^a^	1.53 ^a^
2-octenal	46.8 ^a^	0.97 ^b^
2-pentylfuran	153 ^a^	0.90 ^b^
3-hexen-1-ol	2419 ^a^	10.6 ^b^
butanal	546 ^a^	10.7 ^b^
carbon disulfide	1431 ^a^	11.3 ^b^
decanal	3.70 ^a^	0.64 ^b^
dimethyl disulfide	59.9 ^a^	2.73 ^b^
dimethyl trisulfide	33.2 ^a^	1.95 ^b^
dimethylamine	173 ^a^	121 ^b^
formaldehyde	1443 ^a^	18.0 ^b^
heptanal	85.3 ^a^	2.50 ^b^
hexanal	4566 ^a^	5.60 ^b^
hydrogen sulfide	222 ^a^	0.91 ^b^
methional	7.78 ^a^	0.34 ^b^
methyl mercaptan	1708 ^a^	11.8 ^b^
nonanal	15.5 ^a^	2.10 ^b^
octanal	27.1 ^a^	1.60 ^b^
pentanal	1586 ^a^	32.5 ^b^

**Table A8:** Deodorization of Barley rice protein (BRP) using sequential fermentation. Different letters indicate significant differences within each volatile (*p* < 0.05).

Volatiles	BRP Non-Fermented	BRP Fermented
(E)-2-pentenal	5.49 ^a^	2.37 ^b^
(E,Z)-2,6-nonadienal	1.81 ^a^	0.53 ^b^
1-hexanol	7.89 ^a^	4.25 ^b^
1-octanol	8.16 ^a^	1.31 ^b^
1-octen-3-ol	20.5 ^a^	3.30 ^b^
1-octen-3-one	7.73 ^a^	0.49 ^b^
1-pentanol	37.6 ^a^	25.5 ^a^
1-penten-3-ol	368 ^a^	11.3 ^b^
2,4-decadienal	1.00 ^a^	1.09 ^a^
2,4-heptadienal	203 ^a^	6.76 ^b^
2-heptanone	43.7 ^a^	2.66 ^b^
2-isobutyl-3-methoxypyrazine	1.81 ^a^	0.54 ^b^
2-isopropyl-3-methoxypyrazine	1.70 ^a^	2.08 ^a^
2-octenal	7.24 ^a^	0.54 ^b^
2-pentylfuran	11.8 ^a^	0.49 ^b^
3-hexen-1-ol	193 ^a^	6.67 ^b^
butanal	210 ^a^	6.24 ^b^
carbon disulfide	587 ^a^	5.00 ^b^
decanal	4.78 ^a^	0.44 ^b^
dimethyl disulfide	19.0 ^a^	1.65 ^b^
dimethyl trisulfide	14.1 ^a^	2.69 ^b^
dimethylamine	176 ^a^	84.3 ^b^
formaldehyde	20.8 ^a^	15.4 ^a^
heptanal	135 ^a^	1.49 ^b^
hexanal	375 ^a^	3.24 ^b^
hydrogen sulfide	2.51 ^a^	1.99 ^a^
methional	6.22 ^a^	0.33 ^b^
methyl mercaptan	13.0 ^a^	10.1 ^a^
nonanal	50.4 ^a^	1.15 ^b^
octanal	31.1 ^a^	1.25 ^b^
pentanal	702 ^a^	10.4 ^b^

**Table A9:** Effect of each component of yogurt alternative on the deodorization of off-odor-producing volatile compounds in pea protein. Different letters indicate significant differences within each volatile (*p* < 0.05).

Volatiles	Protein	Protein + Xanthan	Protein + Pectin	Protein + Oil	Protein + *L. plantarum +* Allulose	Protein + *L. plantarum* + Strawberry Preserve	Protein + Traditional Yogurt Cultures + Allulose	Protein + Traditional Yogurt Cultures + Strawberry Preserve
(E)-2-pentenal	13.4 ^c^	17.0 ^b^	16.9 ^b^	19.5 ^a^	8.08 ^e^	9.99 ^d^	16.0 ^b^	13.3 ^c^
(E,Z)-2,6-nonadienal	2.01 ^b^	2.40 ^b^	2.17 ^b^	3.58 ^a^	0.35 ^d^	0.93 ^cd^	0.82 ^cd^	1.33 ^c^
1-hexanol	15.2 ^f^	19.9 ^ef^	27.0 ^cd^	23.9 ^de^	29.4 ^c^	39.3 ^b^	54.4 ^a^	40.2 ^b^
1-octanol	3.02 ^cd^	4.27 ^ab^	3.45 ^bc^	4.60 ^a^	1.73 ^e^	1.77 ^e^	2.99 ^cd^	2.16 ^de^
1-octen-3-ol	40.4 ^c^	54.7 ^ab^	51.7 ^b^	60.2 ^a^	13.6 ^e^	15.5 ^e^	27.6 ^d^	23.3 ^d^
1-octen-3-one	3.77 ^b^	4.36 ^ab^	4.55 ^ab^	5.25 ^a^	1.22 ^c^	1.75 ^c^	2.01 ^c^	1.90 ^c^
1-pentanol	70.7 ^e^	117 ^b^	113 ^b^	129 ^a^	44.5 ^f^	50.3 ^f^	104 ^c^	94.9 ^d^
1-penten-3-ol	372 ^b^	378 ^ab^	400 ^a^	360 ^b^	26.9 ^d^	23.8 ^d^	75.2 ^c^	98.9 ^c^
2,4-decadienal	1.93 ^b^	2.03 ^b^	1.71 ^b^	2.59 ^a^	0.61 ^c^	0.57 ^c^	0.81 ^c^	1.05 ^c^
2,4-heptadienal	337 ^c^	396 ^a^	351 ^bc^	371 ^ab^	48.8 ^e^	18.9 ^f^	73.6 ^e^	176 ^d^
2-heptanone	191 ^b^	184 ^b^	251 ^a^	193 ^b^	68.5 ^d^	79.5 ^d^	135 ^c^	120 ^c^
2-isobutyl-3-methoxypyrazine	3.29 ^b^	5.90 ^a^	2.00 ^bc^	3.32 ^b^	0.82 ^c^	0.82 ^c^	2.45 ^bc^	1.33 ^bc^
2-isopropyl-3-methoxypyrazine	2.82 ^b^	3.47 ^ab^	3.46 ^ab^	3.80 ^a^	1.53 ^c^	1.35 ^c^	1.62 ^c^	1.65 ^dc^
2-octenal	17.7 ^b^	16.7 ^b^	21.7 ^a^	18.0 ^b^	5.86 ^c^	6.64 ^c^	6.14 ^c^	6.34 ^c^
2-pentylfuran	84.2 ^b^	71.2 ^c^	59.4 ^d^	101 ^a^	18.4 ^e^	22.8 ^e^	20.6 ^e^	19.8 ^e^
3-hexen-1-ol	886 ^b^	867 ^b^	940 ^a^	734 ^c^	27.3 ^ef^	18.9 ^f^	71.7 ^de^	117 ^d^
butanal	273 ^ab^	290 ^a^	278 ^ab^	267 ^b^	24.7 ^e^	17.5 ^e^	62.6 ^d^	97.4 ^c^
carbon disulfide	392 ^b^	500 ^a^	504 ^a^	519 ^a^	27.2 ^d^	27.7 ^d^	71.0 ^cd^	97.8 ^c^
decanal	1.50 ^bcd^	2.31 ^a^	2.21 ^ab^	1.87 ^abc^	0.93 ^d^	1.15 ^cd^	1.40 ^cd^	0.78 ^d^
dimethyl disulfide	35.3 ^b^	34.6 ^b^	49.1 ^ab^	62.3 ^a^	10.3 ^c^	11.1 ^c^	39.8 ^b^	32.9 ^b^
dimethyl trisulfide	10.1 ^ab^	9.42 ^ab^	13.2 ^a^	12.3 ^ab^	5.17 ^cd^	4.25 ^d^	9.21 ^ab^	8.89 ^bc^
dimethylamine	62.5 ^c^	60.6 ^cd^	51.8 ^d^	70.1 ^bc^	63.8 ^c^	76.3 ^b^	103.0 ^a^	93.8 ^a^
formaldehyde	111 ^b^	273 ^a^	176 ^b^	23.9 ^c^	12.7 ^c^	16.2 ^c^	32.3 ^c^	22.3 ^c^
heptanal	31.6 ^b^	35.7 ^a^	36.7 ^a^	36.6 ^a^	6.78 ^d^	6.52 ^d^	12.8 ^c^	12.4 ^c^
hexanal	1634 ^b^	1660 ^b^	1784 ^a^	1425 ^c^	46.8 ^e^	21.4 ^e^	111 ^de^	203 ^d^
hydrogen sulfide	24.7 ^b^	76.3 ^a^	31.4 ^b^	2.70 ^c^	1.33 ^c^	0.85 ^c^	1.57 ^c^	1.71 ^c^
methional	2.41 ^c^	3.35 ^ab^	2.96 ^bc^	3.87 ^a^	0.29 ^d^	0.22 ^d^	0.87 ^d^	0.89 ^d^
methyl mercaptan	135 ^b^	325 ^a^	217 ^b^	27.8 ^c^	12.8 ^c^	12.8 ^c^	37.7 ^c^	29.6 ^c^
nonanal	6.76 ^b^	6.74 ^b^	7.81 ^b^	10.7 ^a^	3.01 ^c^	4.26 ^c^	2.48 ^c^	2.30 ^c^
octanal	21.1 ^b^	21.7 ^ab^	25.0 ^a^	23.6 ^ab^	6.06 ^d^	7.36 ^d^	13.2 ^c^	12.1 ^c^
pentanal	752 ^bc^	786 ^ab^	807 ^a^	720 ^c^	26.3 ^f^	20.3 ^f^	93.1 ^e^	144 ^d^

**Table A10:** Effect of co-fermentation versus sequential fermentation on deodorization of off-odor producing volatile compounds in pea protein. Different letters indicate significant differences within each volatile (*p* < 0.05).

Volatiles	Co-Fermentation	Sequential Fermentation
(E)-2-pentenal	11.5 ^a^	5.32 ^b^
(E,Z)-2,6-nonadienal	3.51 ^a^	0.40 ^b^
1-hexanol	19.0 ^a^	17.7 ^a^
1-octanol	4.03 ^a^	1.51 ^b^
1-octen-3-ol	36.8 ^a^	6.44 ^b^
1-octen-3-one	6.83 ^a^	0.85 ^b^
1-pentanol	66.4 ^a^	26.4 ^b^
1-penten-3-ol	203 ^a^	25.1 ^b^
2,4-decadienal	2.12 ^a^	0.70 ^b^
2,4-heptadienal	270 ^a^	12.1 ^b^
2-heptanone	100 ^a^	20.6 ^b^
2-isobutyl-3-methoxypyrazine	4.16 ^a^	1.47 ^b^
2-octenal	20.2 ^a^	2.58 ^b^
2-pentylfuran	81.5 ^a^	8.23 ^b^
3-hexen-1-ol	591 ^a^	26.5 ^b^
butanal	162 ^a^	20.2 ^b^
carbon disulfide	631 ^a^	15.9 ^b^
decanal	5.36 ^a^	1.14 ^b^
dimethyl disulfide	25.2 ^a^	2.22 ^b^
dimethyl trisulfide	9.72 ^a^	1.54 ^b^
dimethylamine	59.5 ^a^	59.9 ^a^
formaldehyde	118 ^a^	9.72 ^b^
heptanal	28.9 ^a^	3.85 ^b^
hexanal	1614 ^a^	42.1 ^b^
hydrogen sulfide	11.9 ^a^	0.97 ^b^
methyl mercaptan	127 ^a^	4.97 ^b^
nonanal	11.3 ^a^	1.98 ^b^
octanal	21.7 ^a^	3.34 ^b^
pentanal	541 ^a^	33.1 ^b^

**Table A11:** Aroma liking, aroma intensity, and off-odor intensity evaluation of non-fermented and fermented protein samples by consumers. Different letters indicate significant differences between the samples within each attribute (*p* < 0.05).

Samples	Aroma Liking	Aroma Intensity	Off-Odor Intensity
Non-Fermented Pea	1.6 ^c^	5.7 ^a^	6.2 ^a^
Fermented Pea	5.4 ^a^	4.3 ^b^	3.4 ^c^
Non-Fermented Soy	3.0 ^b^	4.0 ^b^	4.4 ^b^
Fermented Soy	4.8 ^a^	3.8 ^b^	3.3 ^c^
